# Influence of Fiber Volume in Hybrid Short Glass/Cellulose Reinforced Thermoplastic Compounds

**DOI:** 10.3390/polym14193929

**Published:** 2022-09-20

**Authors:** Christian Kahl, Jan-Christoph Zarges, Hans-Peter Heim

**Affiliations:** Institute of Material Engineering, Polymer Engineering, University of Kassel, 34125 Kassel, Germany

**Keywords:** hybrid reinforcement, regenerated cellulose fiber, fiber content, mechanical properties

## Abstract

Glass fibers (GF) and regenerated cellulose fibers (RCF) are possible partners in the hybrid reinforcement of thermoplastics because of their different properties. Due to the weak bonding properties of polypropylene, coupling agents are used and the fiber volume content is set high to achieve high reinforcing effects. A lower fiber content of GF can raise the toughness properties of a reinforced polypropylene which is investigated in this study with different ratios of GF and RCF. The composites are tested in tensile tests, flexural tests and also in notched Charpy impact tests. The results can be used to compare whether a substitution of GF with RCF or the addition of more GF leads to higher mechanical properties. The tensile and Charpy impact results are compared with the Rule of Hybrid Mixtures (RoHM) to show the deviation to the prediction. Better results in terms of stiffness and strength are seen with a higher total fiber volume, while hybrid reinforced specimens show lower toughness values compared to the RCF reinforced reference specimens. Adding 5 vol% GF to 16 vol% RCF results in an increase in tensile strength by 26%, but also a significant decrease in elongation at break by 65%.

## 1. Introduction

The interaction of two fiber types as reinforcement in a thermoplastic matrix is a suitable method to extend the property spectrum of a matrix [1,2]. A wide range of fiber types available on the market offers extensive possibilities, while GF are the most widely used fibers to reinforce plastics [3].

RCF consist of natural resources, but belong to the chemical fibers due to their manufacturing process, enjoy high popularity due to the sustainability idea and also because of the low density of 1.5 g/cm^3^ compared to a GF with 2.6 g/cm^3^ [4,5,6]. The properties of RCF vary considerably from those of glass fiber [7]. While the glass fiber ensures high modulus and strength in the composite due to its high tensile strength and modulus, the regenerated cellulose fiber offers high ductility in addition to the reinforcing effect due to its high toughness [8].

Since pure GF reinforcement leads to brittleness of the compound with very low elongation at break and impact properties, GF and RCF is a promising combination for hybrid reinforcement [8]. Franciszczak et al. have investigated GF and RCF in PP with different aspect ratios. They reported the best reinforcing effect of the fiber when the aspect ratio is as high as possible. A fiber volume content of 28 vol% was investigated with a ratio of 50/50 (GF and RCF) by volume. The results were compared with reference compounds at the same fiber volume content with only GF or RCF. It was also found that the tensile strength of the hybrid-reinforced compound was below the reference compounds [1].

Romanzini et al. studied glass and ramie fibers with a length of 45 mm by resin transfer molding (RTM) and considered the hybrid effect with different fiber volume fractions. The total fiber volume content in the resin was the determining factor for the mechanical properties in this study. With a brittle matrix system, such as the resin, the impact strength increases with increasing glass fiber content and, in turn, the total fiber volume [9]. In the case of a thermoplastic matrix, such as PP, the addition of fibers leads to an embrittlement of the matrix due to the reinforcing effect. RCF are used in this case to counteract the embrittlement and maintain the toughness of the composite. The mechanical properties of the composite can be controlled by different fiber volume contents, as well as by the ratio of the hybrid components.

For the performance of a fiber-reinforced compound, the bonding of the fiber to the matrix is just as important as the fiber length and also the orientation of the fiber [10,11,12]. In the case of a hydrophobic matrix material, such as polypropylene, coupling agents like maleic anhydrate grafted PP are used to improve the bonding, which results in higher load transfer from the matrix to the fiber. [13]. A RCF should not be bonded well to the matrix, since fiber pull-outs are desired here for high toughness and impact strength. On the other hand well bonded GF gives advantages by load transfer to reach high stiffness and strength [8].

In this study, GF and RCF hybrid fiber reinforced compounds with PP as matrix will be investigated at fiber volume contents of 16 and 21 vol%. The results will be compared with the reference compounds with only one type of fiber. The reference charges are also applied with the Rule of Hybrid Mixtures (RoHM) to make a comparison with the prediction. By using different volume contents, a statement can be made as to whether substitution or addition of glass fibers by regenerated cellulose fibers is appropriate.

## 2. Materials and Methods

### 2.1. Materials

#### 2.1.1. Polymer Matrix

The matrix material used in this study is a polypropylene provided by Sabic (Riad, Saudi Arabia). It is a homopolymer of the type PP 575P. The polymer has a density of 0.905 g/cm^3^ and the melt flow rate is 11 g/10 min at 230 °C and 2.16 kg load.

#### 2.1.2. Fibers

The glass fiber used in this study is the type CS 7952 provided by Lanxess (Cologne, Germany). It is a E-glass fiber with an initial length of 4.5 mm. Due to a silane based sizing it promises a good adhesion to PP. The fibers have a diameter of 14 µm and a density of 2.6 g/cm^3^.

The RCF is a chemical fiber due to its producing process by Cordenka GmbH (Obernburg, Germany) and its starting raw material is bio-based cellulose. The used CR type is made with regenerated cellulose by the viscose process. The fibers have an initial length of 2 mm and a diameter of 12 µm. The density of RCF is 1.5 g/cm^3^.

The mechanical Properties of the fibers from the datasheet are listed in Table 1 [14].

### 2.2. Methods

#### 2.2.1. Compounding

Compounds of PP with GF or RCF were made with a co-rotating twin screw extruder ZSE 18 HPe from Leistritz GmbH (Nuernberg, Germany). The cylinder has a diameter of 18 mm and a process length of 40 D. The temperatures along the seven zones were set from 170 °C to 190 °C (see Table 2). The screw speed was set to 200 rpm along all compounds and the configuration of the screw was chosen to have low shear energy. In this configuration there are only conveying elements after the fibers are fed into the matrix by a side feeder. In that way the shortening of the fibers is less than with configurations with kneading blocks and mixing elements [15]. The throughput was set to 4 kg/h among all compounds. After leaving the nozzle of the extruder, the strand was cooled with compressed air and cut into granulate with a length of 3 mm. Due to their hygroscopic properties, RCF were dried for 24 h at 105 °C in an air convection oven to reach a moisture content of less than 1% before compounding.

The granules with RCF or GF were then mixed to hybrid reinforced compounds in a bag to receive different total fiber volumes and different ratios of the fiber types. Since the fiber addition during compounding is gravimetric, 30 wt% RCF was compounded, which corresponds to a volumetric content of 21 vol%. This fiber content could be used both for the hybrid compounds with 16% and for the reference compounds with 21 vol%.

#### 2.2.2. Injection Molding

**Figure 1:**
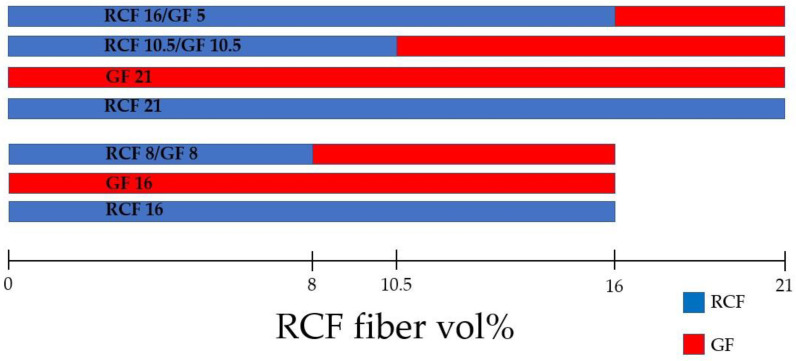
Fiber volume ratios and reference compounds with GF (red) and RCF (blue).

Test specimen of the type 1A according to DIN EN ISO 527 were injection molded with an Arburg (Lossburg, Germany) 320C injection molding machine, which is equipped with a standard three-section screw with a diameter of 25 mm and an open die. It has a clamping force of 50 kN. The temperatures were set from 160 °C to 180 °C and the injection speed to 16 cm^3^/s. The mold temperature was set to 40 °C and the inner mold pressure at injection was 400 bar. Compounds with RCF were dried for 24 h at 80 °C to reach a moisture content less than 0.2 wt%. The specimens were injection molded with to the compounds shown in Figure 1.

#### 2.2.3. Tensile Testing

Tensile tests were carried out on a Zwick Z010 universal testing machine. Attention was drawn to the modulus, the tensile strength and the elongation at break. All specimens were conditioned at 23 °C and 50% humidity for at least 24 h. The tests were carried out according to DIN EN ISO 527. At least 6 specimens were tested of each compound.

#### 2.2.4. Charpy Impact Test

Charpy impact tests according to DIN EN ISO 179 were carried out with a 2 mm deep notch at specimens with a size of 10 mm × 4 mm × 80 mm. The notches were inserted with a device by CEAST GmbH (Martinsried, Germany) with the type A. The specimens were conditioned at 23 °C and 50% humidity for at least 24 h. An instrumented 5 J pendulum hammer was used to record force deformation curves. 8 specimens were tested for each compound.

#### 2.2.5. 3-Point Bending Test

The 3-point bending tests were carried out on a Zwick Roell Z010 according to DIN EN ISO 178. A force drop to 10% of the maximum force was set as the break-off criterion. Flexural modulus, flexural strength and the deformation at the break-off criterion was evaluated.

#### 2.2.6. Rule of Hybrid Mixtures (RoHM)

The RoHM was used to calculate a prediction of the mechanical properties in tensile and impact tests. The formula deals with the reference compounds and the volume of each component [16,17]:

P_H_ = P_1_ ∗ V_1_ + P_2_ ∗ V_2_ (1)

P_H_ represents the property of the hybrid reinforced compounds, P_1_ und P_2_ show the property of compound 1 or 2, which means the property of the reference compounds with GF or RCF in this case. V_1_ and V_2_ show the volume fraction of the compound with PP and fiber 1 or 2. 

#### 2.2.7. SEM Microscopy

The crack surfaces of the tensile and Charpy impact tested specimens were observed using an SEM microscope. Their surfaces were sputter coated with gold before the characterization in the ZEISS Ultra-55 Scanning Electron Microscope (Jena, Germany). The images were obtained using a magnification of 500× to examine the fiber pull-outs, breakages and the fiber distribution in the specimens. The accelerated voltage was 10 kV among all images and an SE detector was used.

## 3. Results and Discussion

### 3.1. Tensile Test

**Figure 2:**
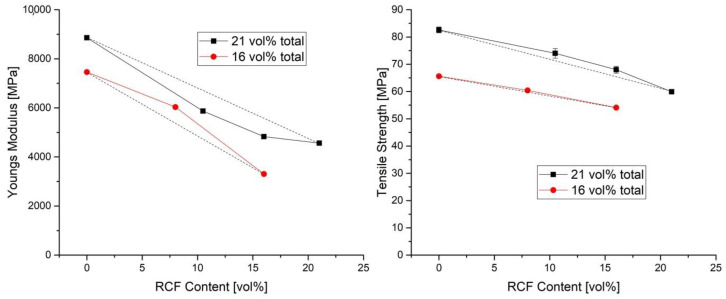
Youngs Modulus (**left**) and Strength of tensile tests (**right**) at 16 and 21 vol% fibers with different type ratios and reference compounds.

**Figure 3:**
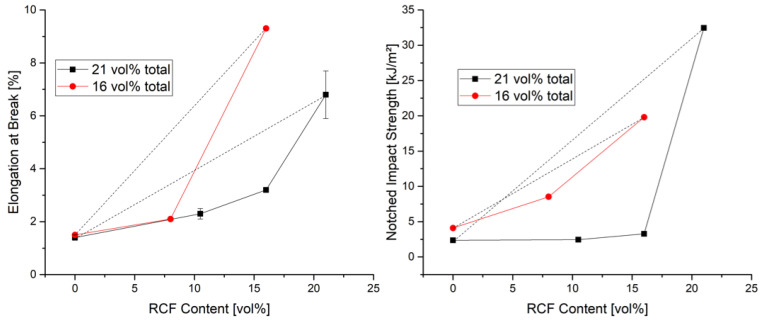
Elongation at Break (**left**) and Notched Impact Strength (**right**) at 16 and 21 vol% fibers with different ratios of GF/RCF and reference compounds.

In this study, fiber volume contents of 16% and 21% were investigated. Reference specimens were prepared with only one fiber type, and the effect of the volume content on the mechanical properties was investigated with both fiber types. The difference in the two volume contents was also used to investigate its effect on the mechanical properties when 5 vol% GF or 5 vol% RCF is added to the reference sample 16% RCF. The results of the tensile tests and the notched impact tests can be seen in Figure 2 and Figure 3.

The prediction is shown as a dashed line between the reference compounds. The measured results show a positive hybrid effect above the dashed line and a negative hybrid effect below the dashed line [18].

Figure 2 shows for the reference compounds, that an increase in fiber volume content results in a significant increase of Young’s modulus and tensile strength. An increase of the GF-content from 16 vol% to 21 vol% offers an increase in E-modulus from 7500 MPa to 8400 MPa, due to more fibers supporting resistance to deformation [19,20]. Also the strength can be increased by a higher fiber volume.

Franciszczak et al. have shown that at a fiber volume content of 28%, the tensile strength of hybrid fiber-reinforced specimens (69.8 MPa) is below the values of the reference specimens with only GF (89.9 MPa) or RCF (74.3 MPa). In this study, the hybrid composites show a clear dependence on the ratio of GF and RCF and the reference compounds (Figure 2). The series with a total of 21 vol% show a negative effect for the hybrid reinforcements and the specimen with 16 vol% a slightly positive hybrid effect. For the strengths, the prediction of the hybrid reinforced compounds properties by the RoHM is accurate. For both, the 16 and 21 vol% total fiber volume content, the results are only slightly above the prediction. 

The RCF16/GF5 compound is the addition of the compound RCF16 with 5 vol% GF, while RCF21 shows the results when 5 vol% RCF is added to the compound RCF16. In terms of strength, the addition of 5 vol% GF means an increase from 54 MPa to 68 MPa (+26%) and for the Young’s modulus an increase from 3305 MPa to 4830 MPa (+46%). 

Figure 3 shows the elongation at break (left) in the tensile test, as well as the Charpy notched impact tests (right). For the elongation at break in the tensile test, it can be noted that the reference compounds with only GF do not give a significant difference and offer an expected low elongation at break compared to the hybrid reinforced and RCF reinforced reference compounds. On the one hand, this effect is due to the good mechanical properties of the GF, but also to a strong bonding of the fiber to the matrix, which results from a silane-based sizing on the surface of the fiber. In the RCF reference compounds, it can be noted that a lower RCF content leads to higher elongations at break. This can be explained by the reinforcing effect of the fiber [21]. A higher content of fiber in the polymer leads to brittleness of the material, which is reflected in a reduction in elongation at break [22,23]. 

The Charpy impact tests according to DIN EN ISO 179 were performed with notched specimens. The notch initiates a crack that propagates rapidly. Due to the impact direction of the pendulum hammer and the bending of the specimen, the specimen is loaded perpendicular to the fiber orientation [24]. Due to the strong embrittlement caused by the GF in the reference compounds, the impact strength is also very low in this test and reaches a value of less than 10 kJ/m^2^ for 16 and 21 vol% fibers. The RCF is pulled out of the matrix with a high energy consumption due to its weak bond to the matrix and the load transverse to the fiber direction [25]. This results in an increase in impact strength with higher RCF contents [26]. More fibers in this case mean more resistance to crack propagation and a higher absorption of energy. Here the impact strength can be increased from 19 kJ/m^2^ at 16 vol% to 32 kJ/m^2^ at 21 vol%.

In the case of hybrid fiber reinforcement, the values of the impact tests show, that at 21 vol% the impact strength remains at the level of the GF reference charge as long as GF is present in the compound. Only the reference compound with RCF has a significantly higher impact strength. Thus, the hybrid effect is negative both in the impact tests and regarding the elongation at break. With 16 vol% fibers, the negative hybrid effect in impact strength is rather small. In this hybrid fiber-reinforced compound (RCF8/GF8), the content of GF is the lowest of all hybrid compounds and, due to the lower fiber volume content, the proportion of impact resistant PP is clearly noticeable here.

A comparison of the two compounds RCF16 and RCF16/GF5 shows in the case of the elongation at break and the impact test a clear decrease of the toughness values by adding a small amount of GF. The embrittlement caused by the GF is so dominant due to the properties of the fiber and its bonding that the high toughness of the RCF cannot counteract this.

### 3.2. 3-Point Bending Test

In contrast to the Charpy impact test, the load in the 3-point bending test is applied slowly to the specimen. Due to the high toughness and the resulting high deformations in the RCF reference specimens, no results could be obtained because the specimens did not fail in this test. For this reason, only the GF reference compounds and one hybrid sample each are evaluated in the 3-point bending test. 

**Figure 4:**
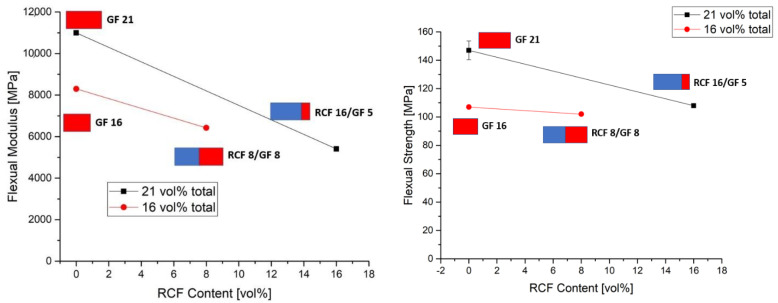
Flexual modulus (**left**) and strength (**right**) of 16 and 21 vol% fibers with different ratios of GF/RCF and GF reference compounds.

As in the tensile tests, the reference charge with only GF shows the highest values of modulus and bending strength (see Figure 4). The high performance was already investigated by Ghanbari et al. [27]. An increase in the total fiber volume from 16 to 21 vol% also leads to an increase in the modulus and strength. If part of the GF is replaced by RCF, the modulus and also the strength decreases. This can also be explained in the 3-point bending test by the good properties of the GF and the good bonding to the PP matrix. Due to this bonding, the forces can be transferred well to the fiber and, in conjunction with the properties of the fiber, provide a high resistance to deformation [28]. The mechanical properties of RCF are below the properties of GF in terms of modulus and strength, which explains a decrease in modulus and strength with increasing RCF content.

**Figure 5:**
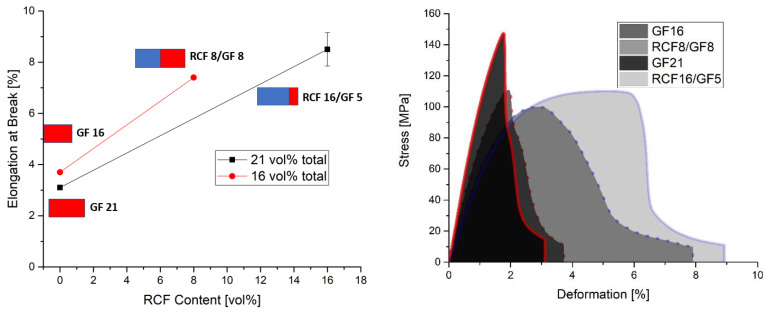
Elongation at flexural break (**left**) and 3 point bending curves (**right**) of 16 and 21 vol% fibers with different ratios of GF/RCF and GF reference compounds.

In addition to the lower properties of modulus and strength compared to the GF, the RCF, however, exhibits a significantly higher toughness (Table 1). Figure 5 (left) shows the results of the strain in the 3-point bending test. Contrary to the previous results, the results of the GF reference compounds are very low due to the reinforcing effect with resulting embrittlement. A low fiber volume fraction of 16 vol% GF leads to a higher elongation than the 21 vol% GF. If half of the GF is replaced by RCF in the 16 vol% fiber content, the elongation increases from 3.8% to 7.4% due to the fiber properties and the fiber bonding. The RCF16/GF5 sample has an elongation of 8.5% compared to the reference GF21 compound of 3.1%. The content of RCF is thus strongly dependent on the toughness of the compound.

The stress deformation curves in Figure 5 (right) of the 3-point bending test also show that the bending stress is highest in the GF reference compounds, but that the specimens fail at a low deformation of approx. 3.5%. As soon as RCF is mixed into the compound, the maximum force decreases at 21 vol% and 16 vol%. However, the deformation increases significantly, indicating that more energy is absorbed, as shown by a larger area under the RCF8/GF8, and RCF16/GF5 curves. 

### 3.3. SEM Images

SEM images of the fracture surface are obtained to identify fiber pullouts and fractures. The surface of both the reference compounds and the hybrid fiber-reinforced compound RCF16/GF5 was examined. The images can be seen in Table 3. Both the tensile test specimens and the impact test Charpy specimens were examined. In the GF reference sample in the tensile test, many fibers can be seen broken just above the surface, which is due to fiber breakage. In this case, a high force has been applied to the fiber, causing it to break. The surface of the impact test according to Charpy shows a higher number of long pulled out fibers which have been pulled out of the matrix due to the direction of the load. The impact strength of this sample is also low compared to the hybrid reinforced and RCF reference samples, indicating that the brittle properties of the fiber lead to low energy absorption. 

Hybrid fiber reinforced specimens also have broken fibers just above the surface after the tensile tests for both RCF and GF. The number of long fiber pullouts is low in the tensile tests. In the Charpy impact tests, the long fiber pullouts increase and can be seen in both RCF and GF. However, due to the high rate of specimen destruction, no appreciable increase in impact strength can be detected. The reference compound RCF shows long fiber pullouts in the tensile tests and also in the impact tests, which can be attributed to a weak bonding of the fiber compared to the glass fibers and the properties in terms of toughness. 

## 4. Conclusions

In this study, reference specimens with RCF or GF were compared with hybrid fiber-reinforced specimens. Two different fiber volume contents of 16 and 21 vol% were considered and tested by tensile tests, 3 point bending tests and Charpy impact tests. The RCF16/GF5 compound was used to investigate the effects of adding 5% GF to the RCF16 compound on the mechanical properties. Based on the results, the following conclusions can be drawn:

- GF reference specimens provide high strengths and stiffnesses in both tensile and 3-point bending tests due to the high properties of the fiber and good bonding to the matrix. An increase in the fiber volume content from 16 to 21% also results in higher strength and stiffness properties.
- For tensile strength, the predictions regarding RoHM are accurate. Only a slight deviation from the prediction can be seen. This applies to both fiber volume contents.
- Toughness values such as elongation at break and Charpy’s impact test show negative hybrid effects at both fiber volume contents. At a fiber volume content of 21%, the impact strength does not increase as long as GF are added to the compound (approx. 2.5 kJ/m^2^). Only the reference specimen with RCF shows a significantly higher impact strength of 33 kJ/m^2^. With the reference sample RCF and a load on the fiber in the tensile direction, a lower fiber volume fraction leads to higher toughness values (elongation at break). Loading transverse to the fiber direction at high speed (impact test) with higher fiber volume contents also leads to higher impact strengths in the RCF reference specimens.
- In the flexural modulus, the stiffness decreases with increasing RCF content. The sample RCF16/GF5 shows a lower stiffness than RCF8/GF8, although the fiber volume content is higher. For flexural strength, the value of RCF16/GF5 is higher than that of RCF8/GF8. Thus, the increasing GF content has an influence on the strength.
- The properties of the compounds can be understood in SEM images. RCF reference samples show very good toughness values in elongation at break and impact strength. This can be proven with long fiber pullouts. The high modulus and strength values in the tensile test and 3-point bending test can be explained by fiber fractures in the GF16 and RCF16/GF5 samples.
- The RCF16/GF5 sample shows that an addition of 5 vol% GF to 16 vol% RCF results in an increase in the mechanical properties in terms of stiffness and strength. This can be seen in the tensile tests as well as in the 3-point bending tests. On the other hand, the addition of GF also results in a significant decrease in the toughness values, which can be seen in the elongation at break in the tensile test and in the 3-point bending test, as well as in the Charpy impact tests.

## Figures and Tables

**Table 1:** Mechanical properties and diameter of RCF and GF.

Short Fiber Type	E-Modulus [GPa]	Strength [MPa]	Elongation at Break [%]	Fiber Diameter [µm]
RCF Cordenka CR Type	22	825	13	12
GF Lanxess CS 7952	73	2600	3.5	14

**Table 2:** Temperature setting along cylinder of double screw extruder.

Zone	1	2	3	4	5	6	7	Nozzle
Temperature [°C]	170	190	190	180	180	180	180	185

**Table 3:** SEM Images of reference and hybrid reinforced specimens after tensile or impact test.

	Impact Test Charpy	Tensile Test
GF21	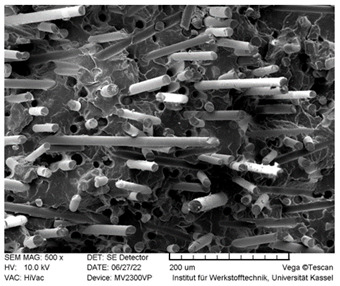	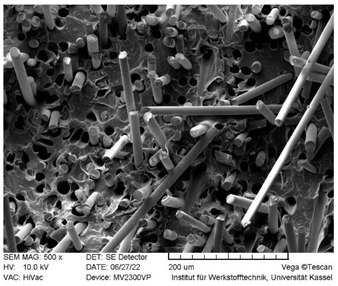
RCF16/GF5	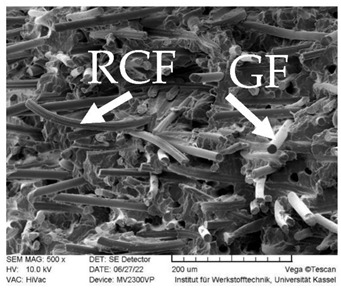	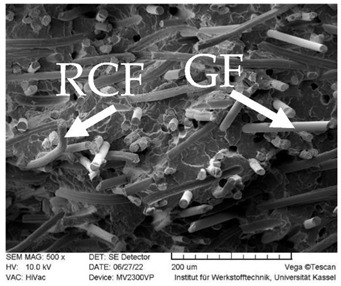
RCF 16	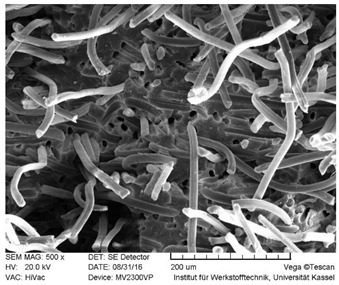	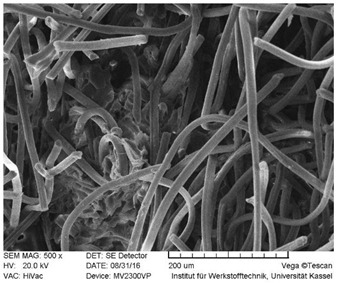

## Data Availability

Not applicable.
